# Detection of EEG K-Complexes Using Fractal Dimension of Time Frequency Images Technique Coupled With Undirected Graph Features

**DOI:** 10.3389/fninf.2019.00045

**Published:** 2019-06-28

**Authors:** Wessam Al-Salman, Yan Li, Peng Wen

**Affiliations:** ^1^School of Agricultural, Computational and Environmental Sciences, University of Southern Queensland, Toowoomba, QLD, Australia; ^2^College of Education for Pure Science, Thi-Qar University, Nasiriyah, Iraq; ^3^School of Electrical and Electronic Engineering, Hubei University of Technology, Wuhan, China

**Keywords:** electroencephalogram, k-complexes, structural undirected graph, fractal dimensions, box counting and time frequency images

## Abstract

K-complexes identification is a challenging task in sleep research. The detection of k-complexes in electroencephalogram (EEG) signals based on visual inspection is time consuming, prone to errors, and requires well-trained knowledge. Many existing methods for k-complexes detection rely mainly on analyzing EEG signals in time and frequency domains. In this study, an efficient method is proposed to detect k-complexes from EEG signals based on fractal dimension (FD) of time frequency (T-F) images coupled with undirected graph features. Firstly, an EEG signal is partitioned into smaller segments using a sliding window technique. Each EEG segment is passed through a spectrogram of short time Fourier transform (STFT) to obtain the T-F images. Secondly, the box counting method is applied to each T-F image to discover the FDs in EEG signals. A vector of FD features are extracted from each T-F image and then mapped into an undirected graph. The structural properties of the graphs are used as the representative features of the original EEG signals for the input of a least square support vector machine (LS-SVM) classifier. Key graphic features are extracted from the undirected graphs. The extracted graph features are forwarded to the LS-SVM for classification. To investigate the classification ability of the proposed feature extraction combined with the LS-SVM classifier, the extracted features are also forwarded to a k-means classifier for comparison. The proposed method is compared with several existing k-complexes detection methods in which the same datasets were used. The findings of this study shows that the proposed method yields better classification results than other existing methods in the literature. An average accuracy of 97% for the detection of the k-complexes is obtained using the proposed method. The proposed method could lead to an efficient tool for the scoring of automatic sleep stages which could be useful for doctors and neurologists in the diagnosis and treatment of sleep disorders and for sleep research.

## Introduction

**FIGURE 1:**
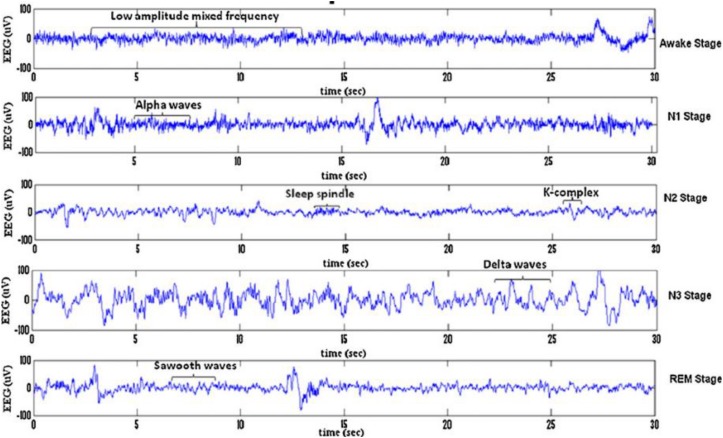
Typical EEG signals of 30 s belonging to sleep stages for a subject: awake stage, N1, N2, S3, N4, and REM stage.

Sleep can be divided into different sleep stages that include mainly non-rapid eyes movements (NREM) sleep, rapid eyes movements (REM) sleep etc. NREM sleep can be further divided into four stages of drowsiness (S1), light sleep (S2), deep sleep (S3) and very deep sleep (S4). Recently, the NREM sleep were reduced by American academy of sleep medicine (AASM) into three stages in which S3 and S4 were combined into one stage as slow waves stages (SWS) (Rechtschaffen and Kales, 1968; Iber et al., 2007; Ranjan et al., 2018). Figure 1 shows the sleep stage signals and their characteristics (Fraiwan et al., 2012). Analysis of these sleep waveforms based on their characteristic features of different stages is an important phase in sleep studies as each sleep stage has different characteristic waveforms. One of those important waveforms occurred in electroencephalogram (EEG) signals and changed over a short time are sleep spindles and k-complexes waves. K-complexes and sleep spindles patterns are the key characteristics of S2, and consequently they are often used to identify S2.

**FIGURE 2:**
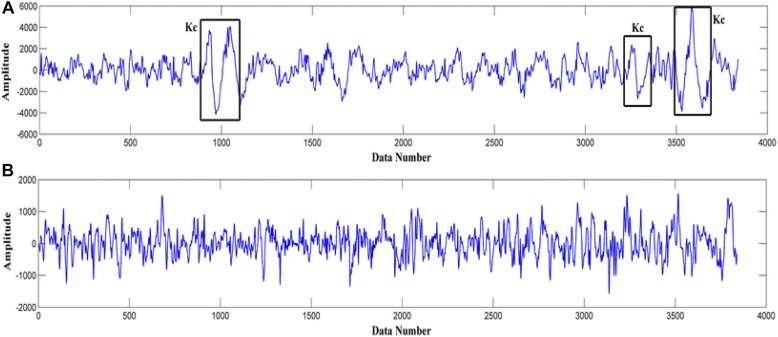
EEG signal examples: **(A)** with k-complexes events. **(B)** without k-complexes.

In 1993 k-complexes were first discovered by Loomis et al. (1938). A k-complex includes a large-amplitude transient waveform with a single negative sharp wave followed by a positive sharp wave, and it has a relatively sharp amplitude that is more than ±75 μV (Bremer et al., 1970; Richard and Lengelle, 1998; Lajnef et al., 2015). This transient bio-signal waveform occurs in all sleep stages, but mainly occurs in sleep stage 2, and it presents in 12–14 Hz waves (Jansen and Desai, 1994). Moreover, in another study (Bremer et al., 1970) it was reported that the minimum peak to peak amplitude value of the k-complexes is around 100 μV. Most of these early studies showed that k-complexes could appear many times during stage 2 with a maximum time duration between 0.5 and 1.5 s. Some studies reported that the maximum time duration of a k-complexes is between 1 and 3 s (Pohl and Fahr, 1995; Lajnef et al., 2015; Hernández-Pereira et al., 2016; Ghanbari and Moradi, 2017; Al-Salman et al., 2018). Examples of EEG signals with and without k-complexes events are shown in Figure 2 (Yücelbaş et al., 2018a).

The k-complexes are very important in both children’s and adults’ sleep studies and the diagnoses of neurophysiologic and cognitive disorders (Bremer et al., 1970; Strungaru and Popescu, 1998; Lajnef et al., 2015). Reliable methods for the analysis and detection of the k-complexes in sleep EEG signals are of great importance for sleep research and clinical diagnosis (Kokkinos and Kostopoulos, 2011). Traditionally, k-complexes are visually examined and marked in an all-night sleep EEG recording by one or two well-trained experts. This process is time consuming, specialist dependent, and tedious, due to the fact that there are typically 1 to 3 k-complexes per minute in stage 2 for young adults (Amzica and Steriade, 2002; Kam et al., 2004; Ghanbari and Moradi, 2017; Ranjan et al., 2018). Therefore, the auto detection of k-complexes is a very important research topic.

In this paper, the fractal dimension (FD) combined with undirected graphs is used to detect k-complexes in sleep EEG signals. Firstly, EEG signal is divided into segments of 0.5 s. Each segment is transformed into a time frequency (T-F) images using a short time Fourier transform (STFT). Secondly, a box counting algorithm is applied to each of the T-F image to calculate their FD. Ten FDs are extracted from each T-F image, and are mapped to undirected graphs to extract the features of interest. The least square support vector machine classifier is used to validate the proposed method. The performance is measured in term of accuracy, sensitivity, and specificity. The performance of the proposed method was compared with several existing methods in the literature. The results demonstrated that the proposed method achieved a high classification accuracy rate for detecting k-complexes in EEG signals.

The remainder of this paper is organized as follows: Section “Related Work” descripts the EEG data used in this paper. Section “EEG Data Description” illustrates the details of the proposed methodology. The experimental results are explained in section “Proposed Method.” Finally, the conclusion is provided in section “Experimental Results.”

## Related Work

Several automatic methods have been developed to detect and analyze the k-complexes. Those approaches used different transformation techniques, such as Fourier transform, wavelet transform, spectral analysis, matching pursuit and autoregressive modeling (Camilleri et al., 2014). So far, no studies have been presented to identify k-complex transient events based on their waveform characteristics, such as a textural descriptor, non-linear features or their graph connections.

Bankman et al. (1992) used a method based on different set of features to detect k-complexes in sleep EEG signals. 14 features were extracted from EEG signals and then used as input into a neural network. The researchers reported an average of sensitivity and false positive rate (FPR) of 90 and 8.1%, respectively. Another study was presented by Hernández-Pereira et al. (2016), in which k-complexes were also detected based on 14 features extracted from each sleep EEG signal. The features were then forwarded to different classifiers to identify k-complexes. An average accuracy of 91.40% was reported using the features selection method.

Tang and Ishii (1995) proposed a method to identify k-complexes based on the discrete wavelet transform (DWT) parameters. The DWT parameters were used to determine the time duration and amplitude of k-complexes. In their study, they obtained 87% sensitivity and 10% FPR. More recently, Lajnef et al. (2015) used a tunable Q-factor wavelet transform for the detection of k-complexes. An average sensitivity and FPR of 81.57 and 29.54% were reported, respectively.

Another study was presented by Richard and Lengelle (1998), in which the k-complexes were recognized based on a joint linear filter in time and time-frequency domains. The k-complexes and delta waves were identified with an average sensitivity and FPR of 90 and 9.2%, respectively. Yücelbaş et al. (2018b) used a method to detect k-complexes automatically based on time and frequency analyses. In their study, an EEG signal was decomposed using a DWT. An average accuracy rate of 92.29% was achieved.

Noori et al. (2014) used a features selection using a generalized radial basis function extreme learning machine (MELM-GRBF) algorithm to detect k-complexes. In their study, fractal and entropy features were employed. The EEG signals were divided into segments using a sliding window technique. The size of the window was set to 1.0 s. An average sensitivity and accuracy of 61 and 96.1% were reported. Researchers in Zacharaki et al. (2013) utilized two steps to detect k-complexes. In the first step, the k-complex candidates are selected, while the number of k-complexes is reduced in the second step using a machine learning algorithm. In that study, four features, including peak-to-peak amplitude, standard deviation, and a ratio of power and duration of the negative sharp wave, were extracted from each segment. An average sensitivity of 83% was reported.

Parekh et al. (2015) detected the k-complexes based on a fast non-linear optimization algorithm. In that study, only F-score result was reported. An average F-score of 0.70 and 0.57% for the detection of the sleep spindles and the k-complexes were achieved, respectively. Another study was presented by Henry et al. (1994), in which the k-complexes were classified based on matched filtering. Each segment was decomposed into a set of orthonormal functions and wavelets analysis.

Devuyst et al. (2010) used a likelihood threshold parameters and features extraction method to detect k-complexes. The performance of the detection was assessed against to two human experts’ scorings. An average of sensitivity rate of 61.72 and 60.94% for scorer 1 and scorer 2 were obtained. Migotina et al. (2010) presented a method based on Hjorth parameters and employed fuzzy decision to identify k-complexes. In that study, the performance of the proposed method was compared with the visual human scoring to evaluate their results. All those methods for classifying k-complexes in sleep EEG signals were based on linear features. So far waveform characteristics based features, such as a textural descriptor, and graph network connections, have not been used for the detection of k-complexes.

According to the literature, we found that the FD as non-linear features has been proven to be an efficient approach to explore the hidden patterns in digital images and signals (Prieto et al., 2011; Finotello et al., 2015). It has been used to analyze and classify EEG signals to trace the changes in EEG signals during different sleep stages, and has also been employed to recognize different digital image patterns. Yang et al. (2007) and Sourina and Liu (2011) employed a FD approach to analyze sleep stages in EEG signals.

Fractal dimension technique was also used by Ali et al. (2016) for voice recognition. Time frequency (TF) images were also used by Bajaj and Pachori (2013) to classify sleep stages. Bajaj et al. (2017) also identified alcoholic EEGs based on T-F images. Based on our previous study (Al-Salman et al., 2018) we found that time frequency images coupled with FD yielded promising results in analyzing and detecting sleep spindles in sleep EEG signals. Furthermore, undirected graph properties have been used to analyze and study brain diseases (Vural and Yildiz, 2010; Wang et al., 2014). Some studies reported that undirected graphs can be considered as one of the robust approaches to characterize the functional topological properties in brain networks for both normal and abnormal brain functioning (Sourina and Liu, 2011; Li et al., 2013). The relevant techniques were employed in image processing as a powerful tool to analyze and classify digital images (Sarsoh et al., 2012).

Recently, a graph approach was used in Diykh et al. (2016) to classify sleep stages. However, in this work, we have combined the fractal features with properties of undirected graphs to detect k-complexes in sleep EEG signals. Based on our knowledge, fractal graph features approach has not been used in k-complexes detection before.

## EEG Data Description

The EEG datasets used in this paper were collected by the Dream project at University of Mons-TCTS Laboratory (Devuyst et al., 2011). The sleep EEG data sets that were publically available included 10 recordings acquired from 10 subjects: 4 males and 6 females using a digital 32-channel polygraph (BrainnetTM system of MEDATEC, Brussels, Belgium) (Devuyst et al., 2010). The sleep EEG data sets were collected in a 30 min interval of the central EEG channel for a whole night. The datasets were sampled at frequency of 200 Hz. Three EEG channels (CZ-A1 or C3-A1, FP1-A1 and O1-A1) and one submental EMG channel were recorded from each subject. The k-complexes in this database were detected visually by two experts. The first expert scored all the ten recordings, while the second expert only annotated five recordings out of the 10 EEG recordings. Therefore, the CZ-A1 channel EEG recordings sampled at 200 Hz, all recording by expert 1, were used for detecting the k-complexes in this study. The information about for the database is shown in Table 1. For more information, please refer to the following website gives details. The dataset with additional information is publicly available from http://www.tcts.fpms.ac.be/~devuyst/Databases/DatabaseKcomplexes.

**TABLE 1:** Database information from dream database.

**Subject ID**	**Sex**	**Age**	**K-complexes scored by expert 1**	**K-complexes scored by expert 2**
ID1	Man	20	34	19
ID2	woman	47	45	8
ID3	Woman	24	12	3
ID4	Woman	23	78	14
ID5	Woman	27	39	20
ID6	Man	23	28	–
ID7	Man	27	11	–
ID8	Woman	46	4	–
ID9	Man	27	5	–
ID10	woman	21	16	–

## Proposed Method

**FIGURE 3:**
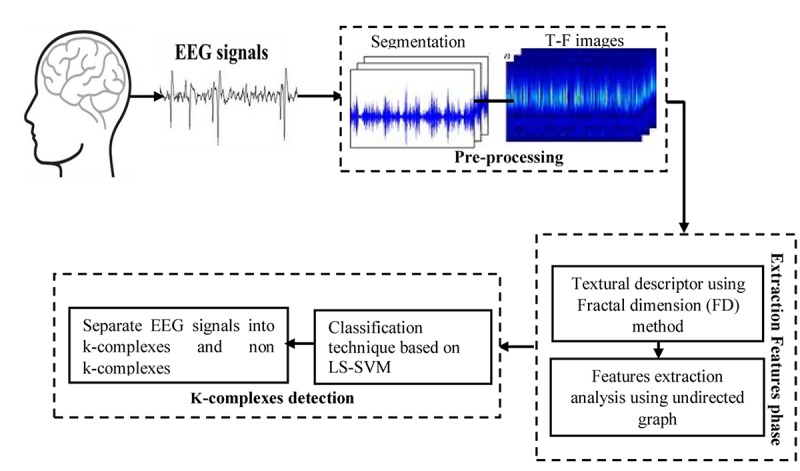
The methodology of the proposed method for k-complexes detection.

In this work, a new method is presented based on time-frequency image and graph features to detect k-complexes in EEG signals. An illustration is given in Figure 3. The EEG signal is firstly divided into segments using a sliding window technique. The size of the window is set to 0.5 s with an overlapping of 0.4 s. Then, each 0.5 s EEG segment is passed through the spectrogram of STFT to obtain the time-frequency images (T-F images). FD as a texture descriptor for each T-F image is calculated based on the box counting method. The vector of FD from each T-F image is then mapped into an undirected graph. Three features of *{degree distributions, Jaccard coefficient, and cluster coefficient}* from each graph are extracted and used as the key features to detect k-complexes in this study. Those features are then forwarded to a least square support vector machine (LS-SVM) classifier to detected k-complexes in EEG signals.

### Segmentation

**FIGURE 4:**
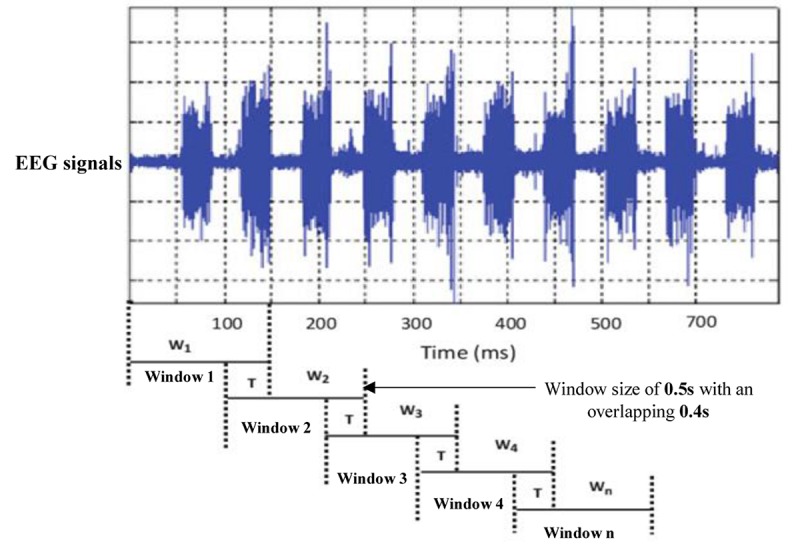
An example of segmenting an EEG signal into windows using a sliding window technique.

Sleep experts have observed that k-complexes normally appear in EEG signals for 0.5 to 2 s. The sliding window technique was utilized by Siuly et al. (2011) for the classification of EEG signals. It was also utilized by Al-Salman et al. (2018) and Zhuang et al. (2016) to detect sleep spindles in EEG signals. Kam et al. (2004) employed the sliding window method to detect k-complexes in their study. Their results showed that applying a sliding window technique helped to improve satisfactory classification results. As sleep spindles and k-complexes occur during stage 2 for about 0.5 to 2 s, we tested various window sizes of 1.0, 1.5, and 2.0 s and overlapping lengths to identify the optimal segment size. However, we made the window length between 0.5 and 2 s. We used the same technique in Al-Salman et al. (2018, 2019). We selected 0.5 window length based on our simulation results. The simulation results showed that the window size of 0.5 s was more optimal for identifying EEG characteristics than other window sizes. Figure 4 shows the EEG signal being dividing into 0.5 s segments with an overlapping of 0.4 s using a sliding window technique.

### Spectrogram of STFT

Spectrogram of STFT is normally defined as the normalized, square magnitude of the STFT coefficient (Bajaj et al., 2017; Al-Salman et al., 2018). The STFT is defined as:

(1) $$S\,\left(n,\omega \right)=\sum_{x=-\infty }^{\infty }y\,\left[x\right]\,w\,\left[n-x\right]\,e^{-j\,w\,n}$$

where *y*[*x*]*w*[*n*-*x*] is a short time of signal *S*(*n*,ω) at time *n*, and the discrete of STFT can be formulated as:

(2) $$S\,\left(n,k\right)={S\,\left(n,\omega \right)|}_{\omega }=\frac{2\,\pi \,k}{N}$$

where *N* refers to the number of discrete frequencies.

Before Fourier transform was calculated, the centered function *w = [x]* at time *n* was multiplied with signal *S*. The Fourier transform is estimated at time *n*, and the window function, *w = [x]* centered at time *n*, of signal *S*(*n*,ω) is considered close to time *n*. A fixed positive function was used to obtain the STFT, which is denoted as *w*[*x*]. Thus, the spectrogram can be formulated as:

(3) $$S\,P\,\left(n,k\right)={\left|S\,\left(n,\omega \right)\right|}^{2}$$

**FIGURE 5:**
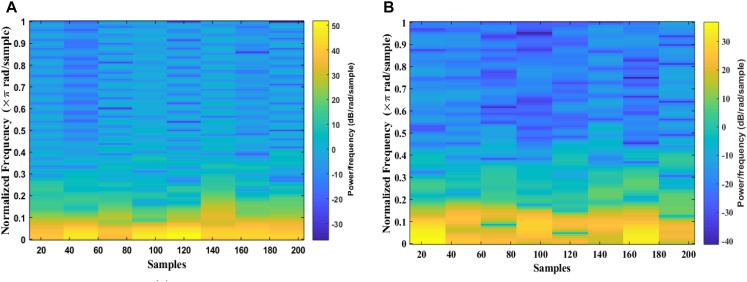
Time-Frequency Image of an EEG segment by the STFT: **(A)** with k-complexes events. **(B)** without k-complexes.

The signal is divided into smaller blocks to obtain the STFT coefficients using the sliding window. After each block is transformed through a Fourier transform, their spectrum is obtained. As the result, the spectrogram of the signal can be calculated from the square of the discrete STFT by using Eqs 1 and 2. Figure 5 shows examples of an EEG segment with a k-complex and an EEG segment without a k-complex event were transformed into a time frequency image using the STFT. According to the literature, the spectrogram is more effective for analyzing non-stationary signals (Siuly and Li, 2012). In this paper, the spectrogram is applied to each EEG segment to obtain the T-F images.

### Fractal Dimension

Fractal dimension allows us to measure the degree of complexity of an object. With FD, each figure can be depicted by a series of fragments. Those fragmented parts can be represented as a small copy of the original figure (Al-Salman et al., 2018).

Extracting features from EEG signals is a common step to obtain the key information. The FD technique is one of the most powerful methods to extract the hidden characteristics from EEG signals (Nunsong and Woraratpanya, 2015) as well as to explore the key patterns in biomedical signals and image processing (Prieto et al., 2011). The FD is commonly used to analyze and classify EEGs signals (Finotello et al., 2015). Based on our previous work (Al-Salman et al., 2018), it was found that extracting features from FD could reduce the complexity of computation time and also increased the detection accuracy.

In this paper, the box counting algorithm is employed and applied to estimate the FD (capacity dimensions) of a T-F image to identify k-complexes in EEG signals. The box counting method can be described as follows: Suppose that **M** is a T-F images and we need to calculate the FD of **M**. The following main formula is utilized.

(4) $$D\,i\,m=\lim_{r\to 0}\frac{\log N\,\left(r\right)}{\log\left(1/r\right)}$$

**FIGURE 6:**
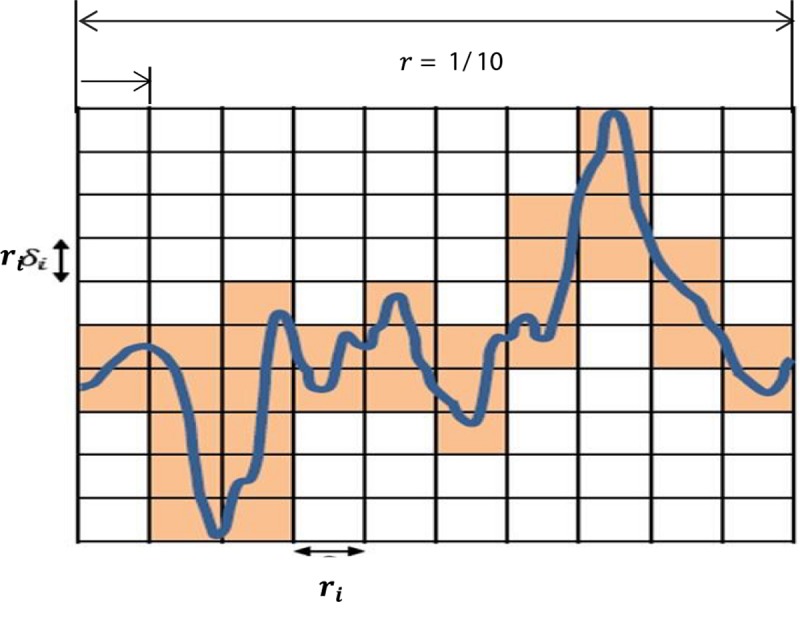
An illustration of the box counting algorithm to create the size and the numbers of boxes.

Based on the equation above, *Dim* is a FD, *N*(*r*) is the total number of boxes, and *r* is the size of boxes that are required to cover image **M**. To cover the entire T-F image, different sizes of boxes are tested, and *N*(*r*) and *r* are determined. Figure 6 presents an example illustrating how the number and size of boxes were created. More details about the box counting algorithm is provided in our previous work (Al-Salman et al., 2019).

### Features Extraction Based on Fractal Graphs

Different window sizes of 0.5, 1.0, 1.5, and 2.0 s were tested in this study to investigate the most suitable number of boxes required to cover the curve. The number of the boxes that are required to cover the entire T-F images using 0.5 s is shown in Table 2, while Table 3 presents the number of boxes with different sizes of windows. As mentioned before, the FD is calculated after transferring an EEG segment into T-F images using the STFT. Then, the box-counting algorithm is applied on each T-F image to extract the features of interest. The values of those features range between 1.0 and 2.0. Each element in the FDs is calculated based on log*N*(*r*)/log(1/*r*). By using the slope of a least square best straight line, the fractal is obtained. From each T-F image, ten FD features as a vector are extracted from each TFI.

**TABLE 2:** The number of boxes in ten scale according to the box size by using 0.5 s window sizes.

**Box size *r***	**1**	**2**	**4**	**8**	**16**	**32**	**64**	**128**	**256**	**512**	**1024**
No. of box *N*(*r*)	277925	70406	17805	6418	1232	360	105	34	12	4	1
log(1/*r*)	0	0.30102	0.60205	0.90308	1.20411	1.50514	1.80617	2.10720	2.40823	2.70926	3.01029
log *N*(*r*)	5.4439	4.8476	4.2505	3.6645	3.0906	2.4857	2.0212	1.5315	1.0792	0.6021	0

**TABLE 3:** The number of the boxes in seven scales using different window size of 2.0, 1.5, 1.0, and 0.5 s.

**Box size *r***	**1**	**2**	**4**	**8**	**16**	**32**	**64**
No. of box *N*(*r*) using 2.0 s	536322	136667	34827	8966	3351	614	168
No. of box *N*(*r*) using 1.5 s	572994	145071	36542	9222	2357	615	168
No. of box *N*(*r*) using 1.0 s	435823	110918	28205	7321	1973	571	166
No. of box *N*(*r*) using 0.5 s	277925	70406	17805	6418	1232	360	105

For example, if the box size *r* is 16, the size of window is 0.5, 1.0, 1.5, and 2.0 s and the number of boxes is 1232, 1973, 2357 and 3351, respectively, as shown in Table 3. Based on the equation of log*N*(*r*)/log(1/*r*), the fractal value for the seventh feature (*FD7*) is 1.204 with window size 0.5 s, as shown in Table 2. However, to obtain 10 FDs from each T-F image, the same procedure is repeated 10 times. In general, the FD values are between 1.0 and 2.0 and all the FD values are non-integer. Based on the experimental results during the training phase, the proposed method provides better classification results using a window size of 0.5 s than the window sizes of 1.0, 1.5, and 2.0 s. More details regarding windows sizes will be presented in section Experimental results.

#### Structure and Construction of Graph Properties

Undirected graph properties have been used to analyze and study brain diseases (Vural and Yildiz, 2010; Wang et al., 2014). The graph may be considered as one of the more robust tools to characterize the functional topological properties in brain networks for both normal and abnormal brain functioning (Stam et al., 2007; Li et al., 2013). It is widely used to identify EEG signals such as sleep stages, as well as to classify digital images (Sarsoh et al., 2012; Diykh et al., 2016). In this study, the structure of graph properties is employed to identify k-complexes from EEG signals.

**FIGURE 7:**
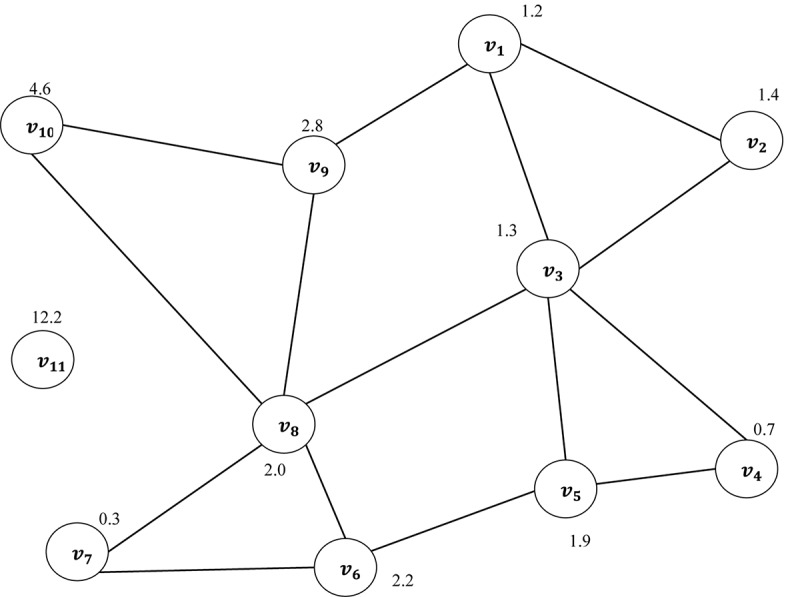
A vector of fractal dimension is mapped into an undirected graph.

An undirected graph can be described as a set of nodes and edges. A graph is a pair of set ***G*** = **(*V, E*)**, where ***V*** is a set of nodes in a graph and ***E*** is a set of connections between the nodes of graphs. Each pair of nodes in a graph is connected by a link. The connection denotes that there are relationships between each pair of nodes in a graph (Blondel et al., 2004; Migotina et al., 2010; Bernhardt et al., 2015). The Euclidean distance has been used in this study as a similarity measure (Huang and Lai, 2006). The edges between the first point and others are calculated using the Euclidean distance. Figure 7 shows a vector of FD as example **X** = {1.2, 1.4, 1.3, 0.7, 1.9, 2.2, 0.3, 2.0, 2.8, 4.6, 12.2, …}, being transferred into an undirected graph which is obtained from the TFIs based on Eq. 4. To construct the undirected graph, each data point in **X** was considered to be a node in a graph. *v*_1_ is the first node in the graph corresponding to the first point in the vector **X** with a value of 1.2. The edges between this point and the others were calculated based on Euclidean distance. More details about Euclidean distance were provided in Zhang and Small (2006), Zhu et al. (2014), and Jain et al. (1999). Consequently, a distance matrix (adjacency matrix) is produced according to Eq. 7. Based on the proposed method, the undirected graph can be characterized with its degree distributions, cluster coefficient and Jaccard coefficient. The next section provides more details in relation to the undirected graph characteristics.

To build the adjacency matrix, we assume that there are two nodes, *v*_1_ and *v*_2_, in an undirected graph. Those nodes are connected if the distance (*d*) between *v*_1_ and *v*_2_ is less than or equal to a pre-determined threshold as explained in the following (Boccaletti et al., 2006; Huang and Lai, 2006; Lacasa and Toral, 2010; Zhu et al., 2014; Diykh et al., 2016).

(5) $$\left(v_{1},v_{2}\right)\in E,\mathrm{if}\,d\,\left(v_{1},v_{2}\right)\leq t\,h\,r$$

where ***thr*** is the pre-determined threshold. Since the structure of the graph is generally biased by the number of existing edges, statistical measures should be calculated on graphs of equal degree ***k***. Therefore, the threshold was defined in this study by adopting the mean degree as an appropriate threshold scheme to reveal the informative network topology which is the average number of edges per nodes of the graph. More details about adopting the mean degree as the threshold was provided in Sporns and Zwi (2004), Stam et al. (2007), Dimitriadis et al. (2009, 2010), and Micheloyannis et al. (2009).

(6) $$k=\frac{1}{n}\,\sum_{i=1}^{n}B\,\left(v_{i},v_{j}\right);n=\mathrm{number}\,\mathrm{of}\,\mathrm{node};$$

Graph ***G*** can be described by giving a square matrix T × T called adjacency matrix *B*. This matrix is used to describe the connection between all the nodes of the graph. The adjacency matrix contains zeros in its diagonal. Thus it is considered to be a symmetrical matrix. The value of this matrix is equal to zero if there is no connectivity among two nodes (*v*_1_ and *v*_1_), and otherwise it is equal to one (Boccaletti et al., 2006). However, the connectivity matrix of an undirected graph is symmetric as *B*(*v_i_*, *v_j_*) = *B*(*v_j_*, *v_i_*).

(7) $$B\,\left(v_{i},v_{j}\right)\,\left\{ \begin{array}{cc} 1, & \mathrm{if}\,\left(v_{i},v_{j}\right)\in E\\ 0, & \mathrm{otherwise} \end{array}\right.$$

It is clear from Figure 7 that the node *v*_11_ of Euclidean distance has no connection to any other nodes in the graph. That means that this node is an isolated point in the graph. In this paper, all the graphs have been constructed with the same number of nodes. The next section provides more details in relation to the undirected graph characteristics.

#### Graph Features

In this study, the adjacency matrix of a graph ***G*** has been used to extract the statistical features. Those statistical features of a graph can be used for the detection of k-complexes from EEG signals in this paper. The following section describes the important features that can be extracted from graph ***G*** (Li et al., 2013; Fang and Wang, 2014; Diykh and Li, 2016).

##### Degree distributions (DD) of the graph

The DD of graph ***G*,** denoted by ***P*(*k*)**, is defined to the proportion of nodes with degree ***k*** partitioned by the total number of nodes in the graph (Stam and Reijneveld, 2007; Zhu et al., 2014; Diykh et al., 2016). It is obtained by counting the number of nodes having degree ***k*** divided by the total number of nodes (Zhu et al., 2014). The DD is defined as:

(8) $$P\,\left(k\right)=\frac{\left|\left\{v|d\,\left(v\right)=k\right\}\right|}{U}$$

where *d*(*v*) refers to the degree of node *v*, while *U* is the total number of nodes in the graph. For example, in Figure 7, $P\,\left(k\right)=\left(\frac{3}{10},\frac{2}{10},\frac{5}{10},\frac{2}{10},\frac{3}{10},\frac{2}{10},\ldots ,\frac{n}{10}\right)$.

##### Clustering coefficient (CC) of the graph

The CC can be considered as one of most important metrics utilized to characterize both local and global structures of a graph, ***G***. It was used by Stam et al. (2007) and Li et al. (2013) to analyze brain activities. Assume that *v_*i*_* is a node in the graph. The clustering coefficient of a given node, *v_*i*_* is calculated as the proportion of the links among *v_*i*_*’s neighbors. For example, the CC of node *v_*s*_* in Figure 7 is 1 as the node *v_*s*_* has three neighbors: (*v*_4_ → *v*_5_, *v*_3_ → *v*_5_, *v*_5_ → *v*_6_). Thus, the CC of *v_*s*_* = 1. The average of the CC of all the nodes is measured as:

(9) $$C\,C=\frac{1}{U}\,\sum_{i=1}^{U}G_{v\,i}$$

where *U* is the number of the nodes in graph ***G*** and *G_*vi*_* is the clustering coefficient of node *v_*i*_*.

##### Jaccard coefficient of the graph

**FIGURE 8:**
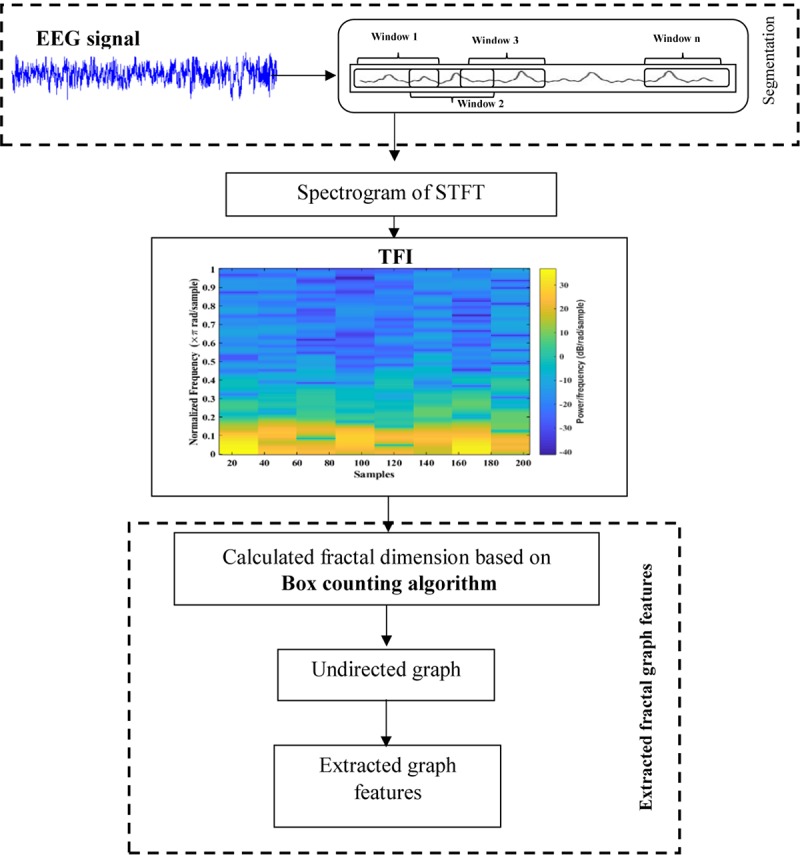
A graphical diagram of feature extraction.

Jaccard coefficient is used to measure the similarity between two nodes of a graph. Assume *v_*i*_* and *v_*j*_* are two nodes in graph ***G***. Jaccard coefficient can be defined as a ratio of the set of the neighboring intersection between *v_*i*_* and *v_*j*_* to the set of the neighboring unions for the two nodes. Jaccard coefficient was used by Anuradha and Sairam (2011) to classify digital image. It was also utilized by Iglesias and Kastner (2013) to analyze the similarity between two time series. Their results showed that using a Jaccard coefficient helped to improve satisfactory classification results. Jaccard coefficient function is calculated based on the following equation:

(10) $$\text{𝐌}\,\left(v_{i},v_{j}\right)=\frac{\left|\Gamma \,\left(v_{i}\right)\cap \Gamma \,\left(v_{j}\right)\right|}{\left|\Gamma \,\left(v_{i}\right)\cup \Gamma \,\left(v_{j}\right)\right|}$$

where Γ(*v_i_*) and Γ(*v_j_*) are the sets of neighbors of the two nods, *v_*i*_* and *v_*j*_*, that have an edge from *v_*i*_* and *v_*j*_*, and 𝐌=[0, 1]. In this study, for each graph, a Jaccard coefficient vector is computed. Figure 8 shows the main steps of the features extraction process using the proposed method.

### Classification Algorithms

After the three fractal graph features are obtained from each graph, they are forwarded to a LS-SVM classifier to identify k-complexes in sleep EEG signals. For comparison, a k-means classifier is also applied. Based on the literature (Siuly et al., 2011; Siuly and Li, 2012; Al Ghayab et al., 2016; Al-Salman et al., 2018, 2019), we found the two classifiers are considered the most popular and effective methods in biomedical signal classification. The training parameters of the selected classifiers were presented in Table 4.

**TABLE 4:** Classifiers’ parameters used in this study.

**Classifier**	**Parameters**
LS-SVM	γ=10, σ = 1 and RBF kernel
K-means	*k*, *c*_*i*_ and *x*_*k*_, where *k* is the number of clusters and *k =* 2. *c*_*i*_ is the center of the clusters and $c_{i}=1$, and *x*_*k*_ is the data points.

#### Least Square Support Vector Machine (LS-SVM)

The LS-SVM classifier was first developed by Suyken and Vandewalle (Guler and Ubeyli, 2007) based on the last version of a support vector machine. It is widely used to classify various types of biomedical signals because it has showed great performance results with a high accuracy rate and low execution time. Many researchers used the LS-SVM classifier to classify different characteristic patterns of EEG signals, such as sleep stages, sleep spindles and epileptic seizures (Sengur, 2009; Siuly and Li, 2012, 2015; Bajaj and Pachori, 2013; Al Ghayab et al., 2016; Diykh et al., 2016). It was used for the detection of sleep spindles in EEG signals in our previous work (Al-Salman et al., 2018).

The LS-SVM classifier generally depends on two hyper parameters, γ and σ. Those parameters should be carefully chosen due to they can positively or negatively affect the performance of a method to increase or decrease the classification rate. The radial basis function (RBF) kernels, γ and σ are empirically selected during the training session. In this paper, the optimum values for γ and σ are set to γ = 10 and σ = 1.

#### K-Means

The k-means classifier is a second classifier being employed in this study. It is considered as one of the most popular approaches in biomedical data classification. In general, the k-means classifier is known as a clustering algorithm (Faraoun and Boukelif, 2006; Al-Salman et al., 2018). It partitions observations into a number of groups according to the similarities or dissimilarities among their patterns. The Euclidean distance for a k-means classifier is usually used for the dissimilarity measure. It was used by Al-Salman et al. (2018) for detecting the sleep spindles, and by Orhan et al. (2011) for detecting the epileptic EEG signals. In this research, the k-means classifier is used to distinguish between k-complexes and non-k-complexes waveforms.

### Performance Evaluation

In order to evaluate the performance of the proposed method with different EEG categories, the following metrics, accuracy, sensitivity and specificity are used in this paper. The main formulas of those statistical measurements are defined as Tawfik et al. (2016) and Yücelbaş et al. (2018b).

(11) $$\begin{array}{c} \mathrm{Accuracy}\,\left(\mathrm{ACC}\right)=\frac{\mathrm{TP}+\mathrm{TN}}{\mathrm{TP}+\mathrm{FN}+\mathrm{FP}+\mathrm{TN}};\\ \mathrm{Sensitivity}\,\left(\mathrm{SEN}\right)=\frac{\mathrm{TP}}{\mathrm{TP}+\mathrm{FN}};\\ \mathrm{Specificity}\,\left(\mathrm{SPE}\right)=\frac{\mathrm{TN}}{\mathrm{TN}+\mathrm{FP}} \end{array}$$

where TN (true negative) is the actual non-k-complexes that are correctly classified as non-k-complexes. FP (false positive) refers to the number of k-complexes that are incorrectly determined by a classifier. TP (true positive) means the actual k-complex waves that are correctly detected. FN (false negative) shows the actual k-complexes that are incorrectly marked as non-k-complexes. More details for those metrics and other measurements are provided in Al-Salman et al. (2018).

#### Matthews’s Correlation Coefficient (MCC)

MCC is used in machine learning as a measure of the quality of binary classifications. It provides a balanced evaluation of the detector as compared with sensitivity and specificity values, which can be used even if classes are of unequal size. It is defined in Migotina et al. (2010) and Matthews (1975):

(12) $$\mathrm{MCC}=\frac{\mathrm{TP}.\mathrm{TN}-\mathrm{FP}.\mathrm{FN}}{\sqrt{\left(\mathrm{TP}+\mathrm{FP}\right)\,\left(\mathrm{TP}+\mathrm{FN}\right)\,\left(\mathrm{TN}+\mathrm{FP}\right)\,\left(\mathrm{TN}+\mathrm{FN}\right)}}$$

#### F-Score

One of the most important measurements that are used to show the overlapping between the two sets. F-score is defined by weighted sensitivity and precision.

(13) $$F-\mathrm{score}=\frac{2\,T\,P}{2\,T\,P+\mathrm{FP}+\mathrm{FN}}$$

#### Kappa Coefficient

It is a statistic measure used to evaluate the agreement between two classification results. In this paper, it is employed to evaluate the agreement between two models, the proposed method and expert (expert 1). It is defined as below:

(14) $$\mathrm{Kappa}\,\mathrm{coefficient}\,\left(k\right)\,\frac{\frac{\mathrm{TP}+\mathrm{TN}}{N}-\mathrm{pre}}{1-\mathrm{pre}}$$

where, $\mathrm{pre}=\frac{\mathrm{TP}+\mathrm{FN}}{N}.\frac{\mathrm{TP}+\mathrm{FP}}{N}+\left(1-\frac{\mathrm{TP}+\mathrm{FN}}{N}\right).\left(1-\frac{\mathrm{TP}+\mathrm{FP}}{N}\right)$, and *N* = (TP + FP + TN + FN).

#### K-Cross Validation

It is a popular approach used for evaluating the performance of a classification algorithm. It is utilized to estimate the quality of the classification results by dividing the number of correctly classified results by the total of the cases. The datasets in section “EEG Data Description” are separated into ***k*** groups with equal size. Each time, one group is used as the testing set, while the remaining subsets (groups) are used as the training set. All the groups are tested in turn. The testing classification accuracy for all groups is calculated. In this paper, 6- cross-validation is used as the accuracy is not improved after *k* > 6. The average accuracy for all testing subsets is computed below:

(15) $$\mathrm{Performance}=\frac{1}{6}\,\sum_{1}^{6}{\mathrm{accuracy}}^{\left(k\right)}$$

where ^(k)^ is the accuracy over the six iterations (*k* = 1, 2, …, 6).

## Experimental Results

**FIGURE 9:**
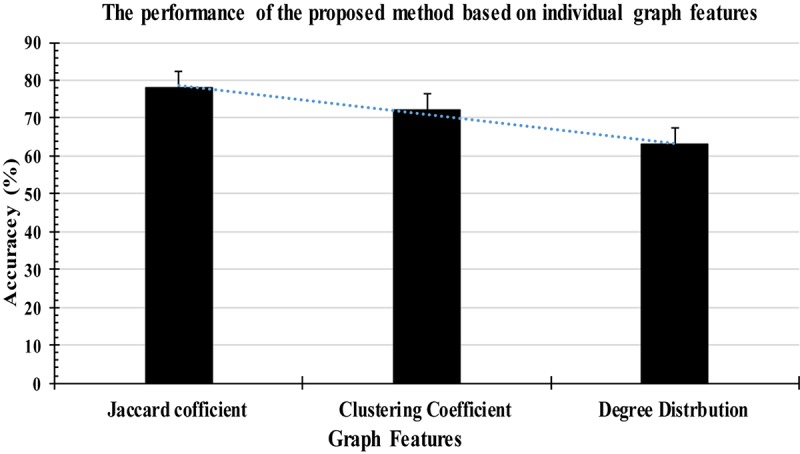
Classification accuracy based on individual graph features.

All the experiments were conducted with the database discussed in section “EEG Data Description” and three structural graph features were extracted from each FD of the T-F images in this study. The features graph were sorted in a descending order based on their importance as shown in Figure 9. Based on the obtained results, the proposed method with the three graph features recorded high classification results, with an average accuracy of 97%. All the experimental results were obtained in a Matlab 2015b environment on a computer that has the following features: 3.40 GH Intel (R) Core^TM^ i7 processor machine, and 8.00 GB RAM. The experimental results were evaluated in terms of accuracy, sensitivity, and specificity. The 6-fold cross validation was also used in this study.

**FIGURE 10:**
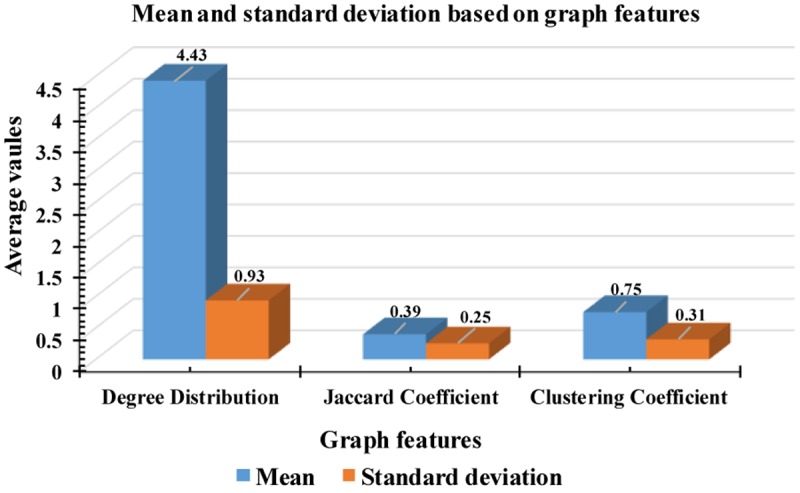
Mean and standard deviation of undirected graph features.

According to Figure 9, some attributes of a graph, such as the Jaccard coefficient, were more significant that other graph attributes in recognizing k-complexes. To investigate the effectiveness of the characteristics of the graph on the identification of the k-complexes, the mean and standard deviation measurements for each segment were used in this study, as shown in Figure 10. From the results in Figure 10, we can see that the three of the graph features: Jaccard coefficient, clustering coefficient, and degree distribution can be used as key attributes to differentiate the k-complexes. All the characteristics of the graph have reported reasonable results in term of standard deviation, as shown on Figure 10. Based on the literature, the obtained results indicate that the three graph features of *{Jaccard coefficient, clustering coefficient, and degree distribution}* can be used to distinguish between k-complexes and non-k-complexes EEG segments.

The results based on the three features set by the proposed method are presented in Table 5. Based on the results in Table 5, it was observed that, the three features set of the graph yields the highest accuracy for the detection of k-complexes in EEG signals. The obtained results demonstrated that the proposed method yielded the best performance with an average accuracy, sensitivity and specificity of 97, 96.6, and 94.7%, respectively. All the results in Table 5 were carried out using LS-SVM classifier with a window size of 0.5 s. For further evaluation, the performance of the proposed method was also tested using a FPR and kappa coefficient. The FPR and kappa coefficient have been calculated for each subject and the average of all the results was investigated. The average of the FPR and kappa coefficient of the proposed method was 0.060 and 0.87, respectively. Based on the literature, the obtained results by the FPR and kappa coefficient provided evidence that the proposed method has the potential to classify k-complexes and non-k-complexes in EEG signals.

**TABLE 5:** The performance of the proposed method based on the DD, JC and CC.

**Fold No.**	**Sensitivity %**	**Specificity %**	**Accuracy %**
Fold1	97	94	98.2
Fold2	96.3	97.8	97.1
Fold3	97.1	96	97
Fold4	97	94	97.3
Fold5	96	92	95.8
Fold6	97	93	96.8
**Average**	**96.6**	**94.7**	**97**

### Performance of the Proposed Method Based on Different Window Sizes

**FIGURE 11:**
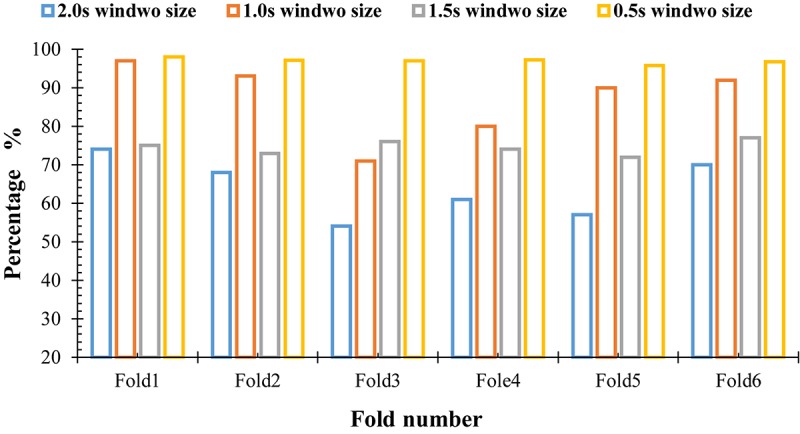
Performance comparisons by the proposed method using different window sizes.

To detect all possible occurrences of the k-complexes in the original EEG signals, and to assess the ability of the proposed method to identify the k-complexes, three other window sizes of 1.0, 1.5, and 2.0 s were tested in this paper. The features described in Section “Graph Features” were extracted, and the dataset was divided into six subsets. The average accuracies of the proposed method were recorded from the 6-fold cross evaluation. The accuracies against the expert’s scoring using different window sizes were reported in Figure 11. From the results in Figure 11, it can be seen that it was difficult to detect k-complexes in EEG signals with 2.0 s window size, which makes sense since the most of the occurrences of k-complexes have a window size of 0.5 s. Our findings show that, there were large disagreements between the proposed method and the expert (Expert 1) in some datasets when 1.5 s window size was used.

On the other hand, it was observed that the proposed method has the capacity to identify k-complexes at a window size of 1.0 s and there was only slight disagreements between the proposed method and the expert’s scoring. Our findings show that the proposed method achieved the highest results when the window size of 0.5 s with overlapping of 0.4 s was used. The maximum accuracy was 97%.

### Performance of the Proposed Method Using Receiving Operating Characteristic Curve

**FIGURE 12:**
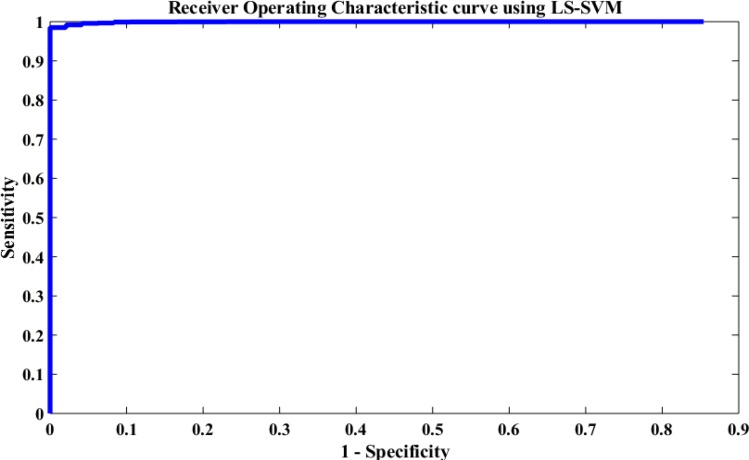
Performance evaluation of the proposed approach using the LS-SVM classifier based on the ROC curve.

The performance of the proposed method was also evaluated based on a Receiving Operating Characteristic (ROC) curve. Figure 12 depicts the ROC analysis results of the LS-SVM classifier. The ROC is a suitable metric in studying the dependence of sensitivity and specificity. The relationship between the true positive rate and FPR were investigated in this paper using the ROC curve. A good test is the one for which sensitivity (true positive rate) rises rapidly and 1-specificity (FPR) hardly increases at all until sensitivity becomes high (Übeyli, 2008). From Figure 12, it is seen that the area value of the ROC curve is 97, which indicates that the LS-SVM model has effectively detected the k-complexes in EEG signals using the extracted features from the graph. Therefore, it is obvious that the fractal graph features well represent the EEG signals and the LS-SVM classifier trained on these features achieves a high classification accuracy.

### Performance Comparisons Using Different Classifiers, Different Data-Driven Thresholding Scheme and With Other Existing Studies

Three types of comparisons were conducted in this section. Firstly, the performance of the proposed method was compared with a different classifier, k-means classifier. Secondly, the proposed method was also compared with different data-driven thresholding scheme. Finally, the proposed method was compared with other studies that used the same datasets as described in section “EEG Data Description.”

#### Comparison With K-Means Classifier

**FIGURE 13:**
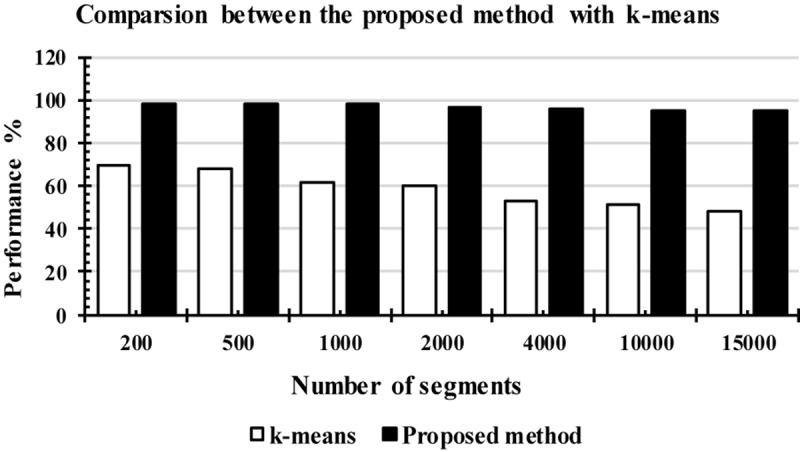
The performance comparison between the proposed method and the k-means classifier.

Figure 13 shows the comparison results between the LS-SVM and k-means classifiers using the extracted features. The same number of segments were used. The segments were chosen randomly from the database. The selected segments were separated into a training set and a testing set, and then were forwarded to the classifiers, separately, to identify k-complexes. Based on the results in Figure 13, it can be observed that the performance of the proposed scheme using the LS-SVM was better than that by the k-means classifier. The accuracy of the k-means classifier was degraded from 65 to 51% when the number of the segments gets to 4000. In terms of accuracy, sensitivity and specificity, the proposed method based on the LS-SVM classifier outperformed the k-means.

**FIGURE 14:**
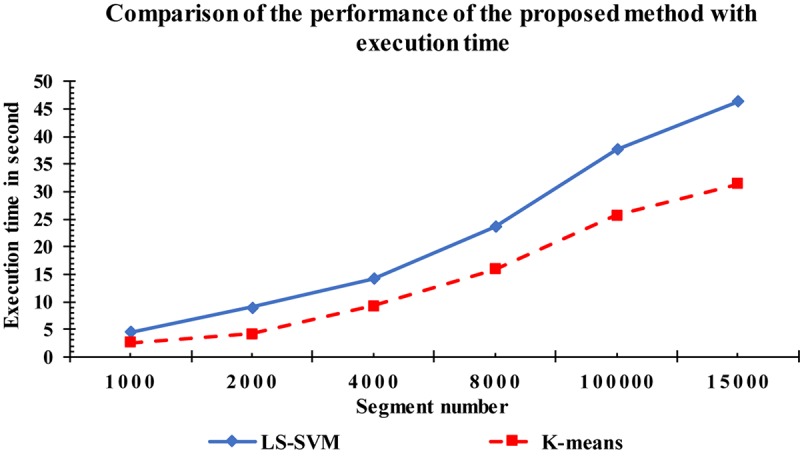
Comparison of the execution time among the proposed method and k-means.

For more investigation, the execution time of the proposed method was calculated based on the LS-SVM classifier as well as to the k-means classifier. Figure 14 shows the complexity time for the LS-SVM and k-means classifiers. To compute the performances of the two classifiers, the same computer having the same settings was used, with the same input data segments. The complexity time of the proposed method was recorded for each classifier. From Figure 14, we observed that the proposed method took an acceptable time although it had more processing steps involved in the algorithm. Based on the obtained results, the highest execution time was recorded with the LS-SVM classifier compared with the k-means classifier. Although converting the fractal features to the undirected graphs take more time, it resulted in more accurate results in k-complexes detection.

**FIGURE 15:**
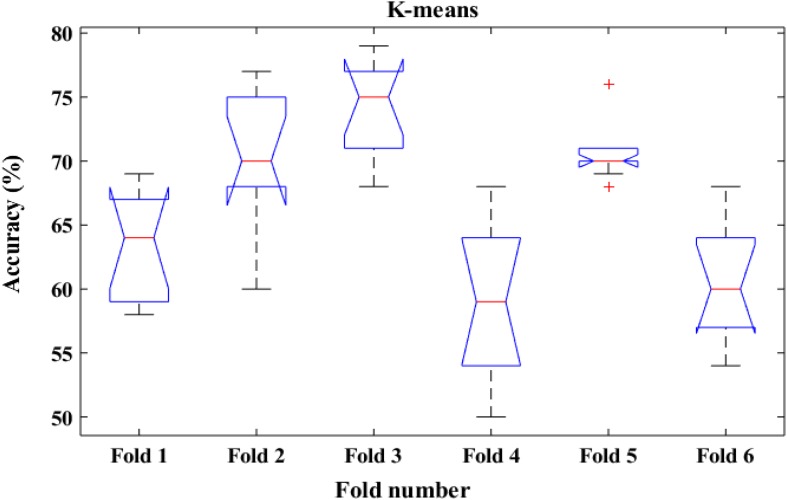
The boxplot of the classification accuracy based on 6-fold cross validation for k-means classifier.

**FIGURE 16:**
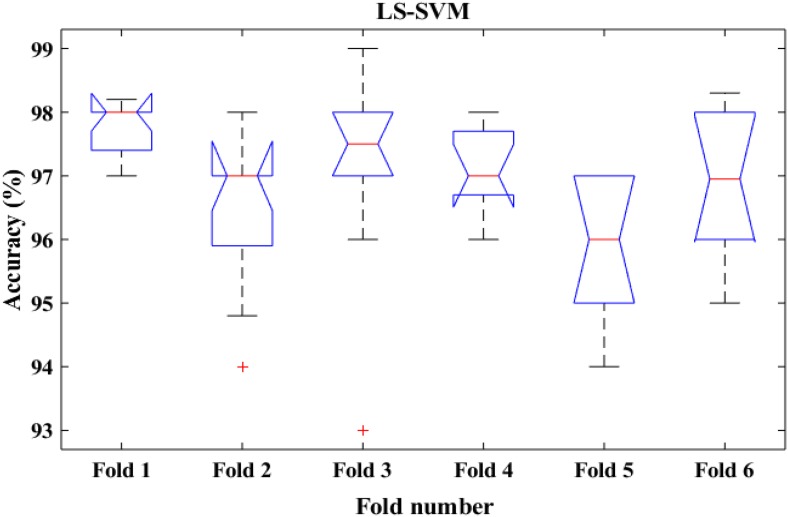
The boxplot of the classification accuracy based on 6-fold cross validation for LS-SVM classifier.

To shed more light on the comparison, the performance of the proposed method was also compared with k-means classifiers for detecting k-complexes in EEG signals based on 6-fold cross validation. The EEG data were divided into six folds and each fold was tested six times. The boxplots for each fold based on 6-fold cross validation were shown in Figures 15, 16. According to the results in Figure 16, it was observed that there was an improvement achieved with the proposed method to detect the k-complexes in EEG signals when the LS-SVM classifier was used to classify the features compared to the k-means classifier. It is clear from these results, the extracted features based on fractal graphs coupled with the LS-SVM classifier have better ability to distinguish the k-complexes in EEG signals.

#### Comparison With Different Data-Driven Thresholding Scheme

The proposed method was tested with different data-driven thresholding scheme reported in Dimitriadis et al. (2017a, b) such as minimal spinning tree (MST) and orthogonal minimal spinning tree (OMST). A spanning tree is a subgraph that includes all nodes of the original graph but it has no cycles. The MSTs try to connect simultaneously all the nodes of the graph by minimizing the cost of the total sum of the weighted links. An MST based on the Kruskal algorithm was used in this study to search the MST in an undirected weighted graph and remove redundant edges. On the other hand, the OMSTs try to capture the most significant connections under the constraint of the MST. More details about the data-drive threshold method was provided in Dimitriadis et al. (2017a, b).

In this paper, the proposed method was also compared with MST and OMST approaches; we optimized the mean degree following a step of 0.1 from mean degree = >5 up to mean degree = <8 toward the maximization of accuracy. The best classification performance was obtained when *k* was 6 and the optimal matching step was 0.2, with an accuracy of 97%, as shown on Table 6. The main reason for that is small mean degrees produces more informative features that further improve classification performance. Also, when the mean degree was small, features that contributed more to the classification were also chosen, leading to higher classification accuracy (Breakspear and Terry, 2002; Rutter et al., 2013; Guo et al., 2018). Thus, the experimental results showed that the optimizing mean degree influenced the classification results. Furthermore, the results in Table 6 indicate that network analysis of an undirected graph to detect k-complexes in EEG signals has been realized in binary graphs using MST, OMST and arbitrary thresholding. However, our findings showed that the proposed method using an arbitrary threshold reported better accuracy, sensitivity and specificity than that of those methods: the MST and OMST. Therefore, in this study, we consider arbitrary thresholding. Table 6 shows the comparison results among different data-driven schemes.

**TABLE 6:** The performance of the proposed method over various thresholding schemes.

**Metrics**	**Types of thresholding schemes**		
	**MST**	**OMST**	**Arbitrary thresholding**
Accuracy	89%	94.6%	**97%**
Sensitivity	91%	95%	**96.6%**
Specificity	94.6%	86.2%	**94.7%**

#### Comparison With Other Methods Based on Different Measurements

**FIGURE 17:**
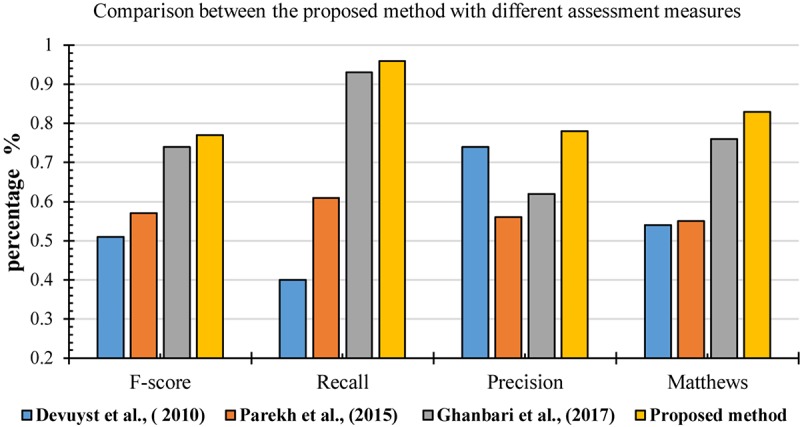
Performance comparison of the proposed method for k-complex detection using different assessment measures.

For further evaluation, the performances of the proposed method was compared with other methods based on different metrics, including F-score, recall, precision and Matthews (MCC). Figure 17 shows the result of comparisons based on different measurements. They were used in different methods to detect k-complexes in EEG signals (Devuyst et al., 2010; Parekh et al., 2015; Ghanbari and Moradi, 2017). They conducted their methods with the same database as used in this study. It can be seen in Figure 17, that the proposed detection approach has a better F-score, recall, precision and MCC values compared with those by other methods. The averages of F-score, recall, precision and MCC were 0.77, 0.96, 0.78, and 0.83%, respectively. Our method performed better than other detection methods, and it achieved higher results compared with those by others.

#### Comparisons With Other Existing K-Complexes Classification Methods

Table 7 represents the performance comparisons among the seven reported methods (Devuyst et al., 2010; Erdamar et al., 2012; Vu et al., 2012; Krohne et al., 2014; Zamir et al., 2015; Patti et al., 2016; Ranjan et al., 2018). All these studies used the same database as discussed in section “EEG Data Description.” According to the results in Table 7, the proposed method is the best among the seven methods. Additionally, it achieved a high accuracy, sensitivity and specificity of 97, 96.6, and 94.7% compared with those methods.

**TABLE 7:** Performance comparisons between the proposed method and other different k-complexes detection approaches with the same datasets.

**Authors**	**Method**	**Accuracy**	**Sensitivity**	**Specificity**
Patti et al. (2016)	Pattern matched wavelets using 400 threshold	–	84%	–
Vu et al. (2012)	Hybrid synergic multi-instance learning machine.	90.2%	70.4%	–
Devuyst et al. (2010)	Likelihood threshold		61.72%	
Ranjan et al. (2018)	Fuzzy algorithm combined with artificial neural network	87.56%	94.04%	76.2%
Zamir et al. (2015)	Convert optimization technique	84%		
Erdamar et al. (2012)	Wavelet transformation combined with a Teager energy operator	91%	89	–
Krohne et al. (2014)	Wavelet transformation	–	74%	–
**The proposed method**	**T-F images coupled with fractal graph features**	97%	96.6%	94.7%

Patti et al. (2016) reported their results of the k-complexes detection with the same database. The average of the sensitivity results they achieved was 84%. The average accuracy was lower than that obtained in this study. Vu et al. (2012) focused on designing a hybrid classifier to detect k-complexes in EEG signals using a hybrid synergic machine learning method. A set of features were extracted from each EEG segment and a representation instance classifier was used to classify the extracted features. Overall, they reported an average of the classification accuracy of 90.2%. Based on the obtained results, the proposed method outperformed the one by Vu et al. (2012).

Another study was made by Devuyst et al. (2010), in which a likelihood threshold was used to detect k-complexes. That study was conducted using the same datasets as the ones used in this paper. The authors reported only true positive rates. The obtained results in our method were higher than those by Devuyst et al. (2010). Ranjan et al. (2018) detected k-complexes using a fuzzy algorithm combined with an artificial neural network. In that study, features were extracted from each EEG segment and then forwarded to a fuzzy neural network algorithm to identify k-complexes in EEG signals. An average accuracy, sensitivity, and specificity of 87.56, 94.04, and 76.2%, were reported, respectively. The classification results were also lower than those by the proposed method. A convert optimization technique was utilized by Zamir et al. (2015) to detect k-complexes. In that study, different features were extracted and ranked based on a feature selection algorithm. The best classification accuracy of 84% was reported. Their accuracy was lower than that of the proposed method.

Erdamar et al. (2012) detected k-complexes using two main stages, including a wavelet transformation combined with a Teager energy operator. In that study, features were extracted based on the amplitude and duration properties of k-complex waveforms. The results from both stages were combined to make a robust method for the detection of k-complexes. In comparison, the proposed method yielded a high classification accuracy than that by Erdamar et al. (2012). Krohne et al. (2014) classified EEG signals into k-complex and non-k-complex segments based on wavelet transformation. In that study, different datasets were used. Their results with both databases were lower than our proposed method. It is clear that the proposed method yielded the highest accuracy compared with the seven other methods using the same datasets.

For further evaluation, the performance of the proposed method was compared with those by Hernández-Pereira et al. (2016), Gala and Mohylova (2009), Ranjan et al. (2018), Noori et al. (2014) based on the types of features and classifiers used. Table 8 shows the results of the comparison. It can be noticed that the proposed scheme reported the highest accuracy compared with the four other methods. The proposed method obtained an average accuracy of 97% with fractal and graph features. This demonstrated that the proposed approach achieved the best performance in terms of classification accuracy.

**TABLE 8:** Comparisons between the proposed method and other studies based on the type of features and classifiers used.

**Authors**	**Features**	**Classifier**	**ACC**
Hernández-Pereira et al. (2016)	12 frequency features.	support vector machine	91.4%
Gala and Mohylova (2009)	Time and frequency domain features	neural network	63%
Ranjan et al. (2018)	12 Bankman features	fuzzy neural network	86.9%
Noori et al. (2014)	Statistic and fractal features	extreme learning machine	96%
**The proposed method**	**Fractal and graph features**	**LS-SVM classifier**	**97%**

## Conclusion

In this paper, the FD technique and undirected graph properties are used to detect k-complexes in EEG signals. In the proposed method, each 0.5 s EEG segment was passed through the spectrogram of the STFT to obtain the time-frequency images (T-F images). Then, the box counting algorithm was applied to each T-F image to calculate the FD. A vector of FD was mapped into an undirected graph to extract the features of interest. Three features were extracted from each graph and they were forwarded to a LS-SVM classifier to identify k-complexes in EEG signals. The experimental results showed that the graph features achieved better performance for the detection of k-complexes with an average accuracy of 97%.

The proposed method was also compared with other existing methods and with different classifiers to identify the ability of using fractal graph features to detect k-complexes. Based on those comparisons the proposed method achieved the best performance in terms of classification accuracy, sensitivity and specificity. The maximum averages of accuracy, sensitivity and specificity obtained using the proposed method are 97, 96.6, and 94.7%, respectively. The outcomes of this study can help the physicians with diagnosing sleep disorders and potentially it can reduce the medical costs. In our future work, the fully weighted version will be taken into consideration as a new methodology to detect other sleep characteristics such as sleep spindles, Sawtooth waves, Alpha waves, and vertex waves.

## Author Contributions

WA-S, YL, and PW contributed conception and design of the study and wrote sections of the manuscript. WA-S organized the database and wrote the first draft of the manuscript. WA-S and YL performed the statistical analysis. All authors contributed to manuscript revision, read and approved the submitted version.

## Conflict of Interest Statement

The authors declare that the research was conducted in the absence of any commercial or financial relationships that could be construed as a potential conflict of interest. The reviewer RLL declared a shared affiliation, with no collaboration, with one of the authors, WA-S, to the handling Editor at the time of review.
